# Severe COVID-19 Associated With Liver Injury in Patients Without Preexisting Liver Disease

**DOI:** 10.7759/cureus.14705

**Published:** 2021-04-26

**Authors:** Abeer Altaf, Zaigham Abbas, Haider A Mandviwalla, Muhammad Ali Qadeer, Mehreen Siyal, Mahnoor Tariq, Asmara Ghafoor, Muniba Karamat, Bushra Shahid, Mahnoor Ali

**Affiliations:** 1 Gastroenterology and Hepatology, Dr. Ziauddin Hospital, Karachi, PAK; 2 Internal Medicine, Dr. Ziauddin Hospital, Karachi, PAK

**Keywords:** covid-19, liver function tests, ast (aspartate aminotransferase), severity of disease, liver injury

## Abstract

Objective: Coronavirus disease 2019 (COVID-19) is known to disturb liver function tests (LFTs). Not much literature is available regarding the effect of COVID-19 on LFTs in patients without preexisting liver disease. The study aimed to find the effect of COVID-19 in these patients.

Methods: This was a single-center, observational study with 142 patients who were admitted with COVID-19 during three months. Seven patients were excluded due to the presence of chronic liver disease.

Results: A total of 135 patients were included in the study aged between 18 and 95 years (mean 57.7 ± 15.6); among them, 93 were males (68.9%). Hypertension was present in 74 patients (54.8%), and diabetes was present in 48 patients (35.6%). Fever was the chief complaint in 112 patients (83%), followed by dyspnea in 93 patients (68.9%) and cough in 79 patients (58.5%). Elevated aspartate aminotransferase (AST) was seen in 35 patients (26%), gamma-glutamyl transferase (GGT) in 43 patients (32%), alanine transaminase (ALT) in 18 patients (24%), alkaline phosphatase in 19 patients (14%), bilirubin in six patients (4%), and low albumin in 27 patients (20%). Severe COVID-19, when compared with mild to moderate disease, was associated with elevated AST > two-time upper limit normal (2ULN) (p = 0.002), GGT > 2ULN (0.026), and lower albumin (p = 0.020), higher systemic inflammatory response syndrome (SIRS) (0.045), raised procalcitonin (p = 0.045), higher ferritin (p = 0.005), lower pO2 (p = 0.044), and higher Sequential Organ Failure Assessment score (SOFA) (p = 0.002) pointing to the inflammatory response as cause of liver injury. Factors predicting mortality with COVID-19 were assessed, which showed that direct bilirubin (p = 0.001), low albumin (p = 0.013), tachypnea (0.002), and leukocytosis (<0.001) were independently associated with increased COVID-19-related mortality.

Conclusion: Patients suffering from COVID-19 have evidence of liver injury, which appears to be secondary to an inflammatory response that correlates with the severity of COVID-19.

## Introduction

The eruption of coronavirus disease-19 (COVID-19) is of great concern globally and has become a substantial challenge for physicians and public health authorities alike [1]. It has affected more than 100 million people worldwide and has been the cause of death of more than two million infected individuals. In Pakistan alone, the number of confirmed cases has surpassed 700,000 [2], crossing a death toll of over 11,000. Although when compared to other countries around the world the number of death is not as high, likely due to the immediate precautions taken by the country’s government from an early stage and the possibility of a large number of infected individuals being asymptomatic [3], the number is still significant enough to be seen as a dangerous amount. The virus’s rapid spread across the globe has largely been due to its alarming rate of infection, primarily through respiratory droplets and secondarily through physical contact [4]. Its usual clinical manifestations in the general population are a dry cough, fever, and flu-like symptoms, which can develop into acute respiratory distress syndrome and multiple system organ failures [5].

Multiple studies have indicated that there is a progression of deranged liver functions in the infected individuals with COVID-19 previously diagnosed to have liver disease [6]. Fewer studies are showing the deteriorating effects of liver function in those without any preexisting liver-related illness. Therefore, it is essential to further investigate these changes in individuals who do not have any prior history of acute or chronic liver disease to better understand the development of abnormal liver functions during the period of infection.

To investigate this further, we documented the derangement of liver function tests (LFTs) among the patients admitted with COVID-19, followed each case, seeing parameters from inflammatory markers to renal functions, coagulopathy, type of admission ward versus ICU, treatment provided, and outcomes.

## Materials and methods

The study was conducted at Dr. Ziauddin Hospital Clifton Campus, Karachi, Pakistan. This was a single-center, observational study. Patients (n = 142) with positive nasopharyngeal COVID-19 PCR for three months were included. Seven patients who had a previous history of chronic liver disease were excluded from the study, leaving a final number of 135. The patients were then followed including the baseline labs, comorbidities, disease course, treatment provided, liver enzymes derangements, and the associations with severity of COVID-19, complications, and outcomes. Disease progress was graded by calculating Sequential Organ Failure Assessment (SOFA) Score, Chronic Liver Failure-Sequential Organ Failure Assessment (CLIF-SOFA) Score, Acute Physiology and Chronic Health Evaluation II (APACHE II) Score, and the number of organ failures. The study was conducted after getting institutional ethics committee approval [2260620ZAGE].

COVID-19 severity was defined as mild, moderate, and severe according to the parameters shown in Table 1.

**Table 1:** COVID severity O_2_, Oxygen; SaO_2_, oxygen saturation; CRP, C-reactive protein; FEU, fibrinogen equivalent units; NLR, neutrophil-lymphocyte ratio; ARDS, acute respiratory distress syndrome.

Mild	Moderate	Severe
No O_2_ requirement > SaO_2_ 93%	O_2_ requirement up to 10L for SsO_2_ 93%	O_2_ requirement >10 L for SaO_2_ 90% and RR > 30
No dyspnea	Dyspnea with <50% of lung involvement	Dyspnea with >50% of lung involvement
No multisystem involvement	No multisystem involvement	Multiorgan dysfunction
D-Dimer < 500 ng/ml FEU	D-Dimer 500-1000 ng/ml FEU	D-Dimer > 1000 ng/ml FEU
CRP < 5 mg/L	CRP 5-10 mg/L	CRP > 10 mg/L
Ferritin < 500 ng/ml	Ferritin 500-1000 ng/ml	Ferritin > 1000 ng/ml
NLR=1	NLR < 3	NLR > 3
No secondary infection	No secondary infection	Evidence of secondary infection
No ARDS	ARDS SpO_2_/FiO_2_ 200-300	ARDS < 200
No shock/need for inotropes	No shock/need for inotropes	Shock/need for inotropes

Continuous variables were expressed as mean and standard deviation, and analysis was performed by Mann-Whitney U test. Categorical data were analyzed using Fisher's exact test. A p-value of <0.05 was considered statistically significant. The statistical analysis was done on the IBM SPSS software version 20.0 (IBM Corp., Armonk, New York).

Acute liver injury was defined as a patient with any one of the following [7]:

 i. Jaundice, with total bilirubin > 3 mg/dl,

 ii. Liver enzymes, alanine transaminase (ALT), aspartate aminotransferase (AST), gamma-glutamyl transferase (GGT), and alkaline phosphatase (ALP) > two-time upper limit normal (2xULN),

 iii. International normalized ratio (INR) > 1.5, with no previous history of liver dysfunction.

## Results

A total of 135 patients of age between 18 and 95 years (mean = 57.68) were included among which 93 were males (68.9%) and 42 were females (31.1%); 107 patients (79.25%) improved and were discharged home, and 28 patients died (20.7%). Of the 135 patients, 57 had mild symptoms (42.22%), 32 had moderate symptoms (23.7%), and 46 (34.07%) suffered from severe COVID-19. The mean age of individuals affected with severe COVID-19 was 60.70, and the mean age of individuals affected by mild to moderate disease was 55.38. Mortality was highest in individuals admitted with severe COVID symptoms who required critical care (92.85% vs 7.14%)

Among the comorbid conditions in the patients, hypertension was present in 74 patients (54.8%), diabetes was present in 48 patients (35.6%), cardiovascular disease was present in 25 patients (18.5%), chronic kidney disease was present in seven patients (5.2%), chronic obstructive pulmonary disease was present in six patients (4.4%), and neurologic diseases were present in five patients (3.7%). Of the study population, seven (5.2%) were smokers, four of them had a history of alcohol intake (3%), and obesity was present in six (4.4%) individuals. Preexisting drug history included beta-blockers in 21 patients (15.6%), diuretics in 35 patients (25.9%), angiotensin-converting enzyme inhibitors in 31 patients (23%), nonsteroidal anti-inflammatory drugs (NSAIDs) in eight patients (5.9%), and statins in 20 patients (14.9%).

The chief complaint of the majority of individuals (112 patients) admitted with COVID-19 was fever (82.96%), followed by shortness of breath in 93 patients (68.9%), cough in 79 patients (58.5%), body aches in 27 patients (20%), nausea in 10 patients (7.4%), vomiting in nine patients (6.7%), sore throat in seven patients (5.2%), and two individuals presented with hematemesis (1.5%). The presence of clinical or laboratory-proven jaundice was seen in one patient (0.7%).

Liver enzyme derangements were seen in 32% of patients admitted and treated for COVID-19 and correlated with disease severity and outcome as evident in Table 2. AST levels ranged from 13 to 1883, a mean of 107.47 ± 212.70. Levels more than two times the ULN were seen in 23 patients with severe COVID-19 (p = 0.002). ALT levels ranged from seven to 1762, a mean of 95.1 ± 199.62. Levels more than two times the ULN were seen in 16 individuals with severe COVID-19 (p = 0.096). GGT levels were between 12 and 964 with a mean of 121.24 ± 135.24. Levels of 2xULN were seen in 28 individuals suffering from severe COVID-19 (p = 0.026). Total bilirubin ranged between 0.2 and 15.2 with a mean of 0.95 ± 1.72. Values above 2 mg/dl were present in three patients with severe disease (p = 0.302). ALP values were between 11 and 249 with a mean of 91.59 ± 44.54. ALP more than 120 were seen in 10 patients with severe disease (p = 0.094).

**Table 2:** LFTs in Severe COVID ALT, Alanine transaminase; AST, aspartate aminotransferase; GGT, gamma-glutamyl transferase; ALP, alkaline phosphatase; INR, international normalized ratio; 2xULN, two-time upper limit normal; LFT, liver function test.

Liver Function Tests	Mild to Moderate COVID-19		Severe COVID-19		P-value
	Mean ± SD	Range	Mean ± SD	Range	
Total bilirubin (mg/dl)	0.675 ± 0.42	0.2 - 2.4	1.41 ± 2.73	0.3 - 15.2	0.120
ALT (U/L)	86.56 ± 213.06	7 - 1762	110.05 ± 175.21	7 - 1061	0.116
AST (U/L)	93.22 ± 218.61	15 - 1883	131.65 ± 202.49	13 - 1288	0.022
GGT (IU/L)	103.65 ± 103.2	12 - 586	150.70 ± 173.84	14 - 969	0.041
ALP (U/L)	88.41 ± 42.09	11 - 249	96.98 ± 48.45	35 - 220	0.058
Albumin (g/dl)	3.82 ± 0.47	2.5 - 4.7	3.49 ± 0.69	1.9 - 4.6	0.002
INR	1.18 ± 0.20	0.9 - 2.0	1.35 ± 0.41	0.9 - 2.7	0.042
Elevated ALT ≥ 2xULN	43 ± 50.91	7 - 79	570.5 ± 693.67	80 - 1061	0.096
Elevated AST ≥ 2xULN	47 ± 45.25	15 - 79	684 ± 604	80 - 1288	0.002
Elevated GGT ≥ 2xULN	45.5 ± 47.37	12 - 79	524.5 ± 628.61	80 - 969	0.026
Albumin < 3.5 g/dl	4.105 ± 0.84	3.51 - 4.7	2.695 ± 1.124	1.9 - 3.49	0.002

Derangement of AST with more than two times ULN (>80) was compared with both mild/moderate and severe COVID as shown in Table 3. AST > 80 was significant for higher severe inflammatory response syndrome (SIRS) score 1.29 ± 0.463 vs 1.59 ± 0.503 (p = 0.045), raised procalcitonin 0.07 ± 2.15 vs 9.24 ± 21.12 (p = 0.045), raised ferritin 1008.14 ± 1327.92 vs 2354.89 ± 2567.03 (p = 0.005), decreased oxygen in arterial blood gases on admission 91.75 ± 33.91 vs 71.77 ± 32.88 (p = 0.044), higher SOFA score 1.72 ± 1.52 vs 4.0 ± 2.50 (p = 0.002), and increased number of organ damage 0.88 ± 0.69 vs 1.75 ± 0.86 (p = 0.006). Outcomes of the patients were also monitored and showed that 51 patients with AST < 80 improved and 12 died. In patients with AST > 80, 22 were improved and sent home, whereas 16 passed away (p = 0.021).

**Table 3:** AST > 2ULN and severity of COVID SIRS, Systemic inflammatory response syndrome; WBC, white blood cell; PCT, procalcitonin; CRP, C-reactive protein; SOFA, Sequential Organ Failure Assessment; APACHE II, Acute Physiology and Chronic Health Evaluation II; AST, aspartate aminotransferase; 2ULN, two-time upper limit normal.

Variable	AST < 80		AST > 80		P-value
	Mean	SD	Mean	SD	
Mean arterial pressure	91.97	12.17	90.59	15.43	0.631
PaO_2_/FiO_2_	108.82	143.15	98.18	184.13	0.804
SIRS	1.29	0.463	1.59	0.503	0.045
Hb	12.17	2.38	13.41	1.52	0.01
WBC	10.71	4.95	10.78	4.95	0.943
Neutrophils	77.82	11.509	81.74	8.98	0.075
Lymphocytes	15.57	9.994	13.18	8.037	0.213
Platelets	244.03	121.80	209.83	92.88	0.127
Creatinine	1.37	1.15	1.27	0.71	0.61
PCT	0.97	2.15	9.24	21.12	0.045
CRP	113.30	132.87	121.26	55.75	0.72
Lactate	1273.89	2696.26	40.27	41.85	0.33
Ferritin	1008.14	1327.92	2354.89	2567.03	0.005
D-Dimer	1775.53	2600.94	2663.31	3385.07	0.150
PaO_2_	91.75	33.91	71.77	32.88	0.044
SOFA	1.72	1.526	4.00	2.50	0.002
APACHE II	8.44	5.56	10.43	7.01	0.340
No. of organ failure	0.88	0.69	1.75	0.86	0.006

COVID-19 severity itself was also studied, and it was seen that patients suffering from severe illness have higher white blood cell (WBC) levels 9.97 ± 3.97 vs 11.69 ± 5.46 (p = 0.04), neutrophilia 76.65 ± 10.18 vs 82.91 ± 9.21 (p = 0.001), lymphopenia 16.62 ± 8.99 vs 11.8 ± 7.47 (p = 0.003), and hence a higher neutrophil to lymphocytic ratio. Patients also had higher creatinine 1.21 ± 0.66 vs 1.58 ± 1.25 (p = 0.023), and some even required renal replacement treatment. LFTs such as AST 65.60 ± 51.23 vs 130.63 ± 207.14 (p = 0.022) and GGT 94.15 ± 80.88 vs 156.02 ± 176.36 (p = 0.041) were also significantly deranged, and often patients developed hypoalbuminemia as well 3.89 ± 0.44 vs 3.57 ± 0.503 (p = 0.001). Labs like INR 1.15 ± 0.169 vs 1.28 ± 0.29 (p = 0.042) and D-dimers 1093.93 ± 1397.4 vs 3001.3 ± 3824.8 (p = 0.003) also showed substantial derangements. Prognostic markers of severity such as procalcitonin, lactate, C-reactive protein (CRP), and ferritin were also significantly elevated among the severely affected population with ferritin being statistically significant among all 1047 ± 1258.46 vs 17779.67 ± 1931.84 (p = 0.017). Disease severity calculators such as APACHE II Score 5.33 ± 2.24 vs 12.83 ± 6.95 (p < 0.001) and SOFA Score 1.5 ± 1.15 vs 4.3 ± 2.43 (p < 0.001) were also higher in severe patients as compared to mild to moderate ones. Arterial oxygen levels were also low in these patients on admission 89.62 ± 9.81 vs 93.28 ± 3.86 (p = 0.002), and they hence required supplemental oxygen via face mask, high-flow nasal cannula (HFNC), noninvasive ventilation (NIV), or ventilator.

Factors predicting mortality with COVID-19 were also assessed as mentioned in Table 4. Stepwise linear regression analysis of continuous variables showed four variables such as direct bilirubin (p = 0.001), low albumin (p = 0.013), increased respiratory rate (0.002), and raised WBC count (<0.001), which were independently associated with increased COVID-19-related mortality.

**Table 4:** Factors associated with mortality TLC, Total leucocyte count; ALT, alanine transaminase; AST, aspartate aminotransferase; GGT, gamma-glutamyl transferase; ALP, alkaline phosphatase; INR, international normalized ratio; PCT, procalcitonin; LDH, lactate dehydrogenase; 2xULN, two-time upper limit normal; LFT, liver function test; DIC, disseminated intravascular coagulation.

Variable	Alive	Dead	P-value
	Mean/N (SD/%)	Mean/N (SD/%)	
Age	55.96 (15.91)	63.30 (12.85)	0.21
Mean arterial pressure	91.59 (13.863)	87.00 (17.75)	0.103
Respiratory rate	22.75 (3.55)	25.59 (6.09)	0.002
SpO_2_	93.05 (5.79)	87.28 (11.13)	<0.001
TLC	9.82 (4.30)	13.37 (5.28)	<0.001
Neutrophils	77.44 (11.06)	83.89 (8.29)	0.005
Urea	48.32 (47.31)	60.07 (44.46)	0.033
Creatinine	1.16 (0.81)	1.87 (1.43)	0.001
Total bilirubin	0.63 (0.39)	1.80 (3.20)	0.001
Direct bilirubin	0.29 (0.196)	1.19 (2.44)	0.001
ALT	87.35 (199.209)	117.79 (202.761)	0.489
AST	90.05 (201.79)	157.40 (237.87)	0.136
GGT	115.36 (136.86)	137.90 (131.35)	0.435
ALP	87.71 (42.04)	102.70 (50.13)	0.113
INR	1.18 (0.18)	1.44 (0.47)	0.020
PCT	1.14 (5.40)	12.37 (22.76)	<0.001
Fasting blood sugar	156.65 (74.67)	113.45 (10.92)	0.062
LDH	864.41 (507.90)	1207.72 (497.25)	0.003
D-dimer	1357.56 (2240.19)	3562.42 (3551.39)	<0.01
SOFA score	1.89 (1.39)	5.11 (3.19)	<0.001
APACHE II score	6.50 (3.85)	14.17 (6.44)	<0.001
No. of organ failure	0.96 (0.64)	2.00 (0.77)	0.004
Fever	92 (82.14%)	20 (17.85)	0.117
Shortness of breath	74 (79.56%)	19 (20.43)	1.00
Cough	67 (84.18%)	12 (15.18%)	0.298
Presence of clinically significant jaundice	0 (0%)	1 (100%)	0.043
Severe COVID-19	20 (41.67%)	28 (58.33%)	<0.001
Albumin < 3.5	22 (73.68%)	16 (42.10%)	0.013
AST > 80	57 (82.60%)	12 (17.39%)	0.009
Acute kidney injury	5 (27.77%)	13 (72.22%)	<0.001
Respiratory failure	14 (42.42%)	19 (57.57%)	<0.001
Derangement in LFT	9 (56.25%)	7 (43.75%)	0.043
Hemodynamic shock	1 (6.25%)	15 (93.75%)	<0.001
DIC	10 (58.82%)	7 (41.17%)	0.059

## Discussion

Acute liver injury is defined as a derangement in LFTs due to an acute insult in a previously healthy liver. Many studies have reported patients developing liver injury following COVID-19 infection [7-9]. Reasons for this liver injury are still unsure, but multiple studies show it may be due to the expression of angiotensin-converting enzyme II (ACE-II), which is identified as the main receptor for COVID-19 entry into cells. This could suggest that COVID-19 could have a direct effect on the hepatic system along with a sequalae to the ongoing inflammatory response [10,11]. This could also be due to the difference in population and how ACE-II expression analysis differs between ethnicities [12], but further studies are required. Another hypothesis suggests immune-mediated liver injury, i.e., cytokine storm, causing the release of multiple pro-inflammatory cytokines particularly IL-6 that causes both pulmonary and extrapulmonary damages including the liver [8]. Patients affected with severe disease often have hypoxia and hypotension, and that might also be a causative factor to cause acute liver injury [13]. Lastly, many of the critically ill patients are on a variety of medications and parenteral nutrition, which may also contribute to the ongoing liver insult. All of these are the most commonly highlighted reasons for causing both direct and indirect hepatic damage in COVID-19-affected population.

As hypertension was the most common comorbidity in patients within our study, drug history might be significant in being a cause of worsening deranged liver enzyme levels. The three most common medications patients were using before admission were diuretics (25.9%), angiotensin-converting enzyme (ACE) inhibitors (23%), and beta-blockers (15.6%), followed by statins (14.9%). There is not enough evidence to prove whether the indication of using these drugs before hospitalization is truly significant to deranging liver enzymes [14].

The mean values of LFTs were above the normal range as seen within this study (Table 2). The liver function enzyme levels of patients with severe cases of COVID-19 were more deranged as compared to the liver enzyme levels of patients with mild or moderate cases. These enzymes, when elevated together, are strong indicators of liver injury. Total bilirubin (TB) and ALP levels were elevated in only a few cases, but overall remained under the normal ranges (mean TB = 0.95, mean ALP = 91.59). These markers in numerous studies have had different results, some in which TB is markedly increased [15,16] and others that coincide with our study [17]. Serum albumin levels were also not severely affected, but a mean decrease was seen in severe cases (Alb = 3.49), which is closer to the lower normal limit as compared to the mild/moderate cases (Alb = 3.82).

AST levels greater than 80 showed more deranged infective markers typically seen in COVID-19 cases as shown in Table 3. AST > 80 showed severely deranged results (mean lactate = 2696.26, mean D-dimer = 2663.31, mean ferritin = 2354.89, mean PCT = 9.24). Although these markers were not as severely deranged in patients with AST < 80, they were deranged nonetheless, showing how this presence of injury to the liver could be secondary to an inflammatory response. This has been seen in multiple studies within our subcontinent [17] and across the globe [18].

SOFA and APACHE II scores were also calculated to assess the prediction of mortality and to cross-reference with the severity of liver enzyme derangement. In severe cases, SOFA and APACHE II scores were markedly raised (mean SOFA = 4.33, mean APACHE II = 12.83), almost three times higher as compared to the scores calculated in mild/moderate cases. These calculations, including the significant increase in infective markers and LFTs, may show an overall systemic effect by the COVID-19 virus and may indicate a positive correlation between an increase in viral, liver injury, and inflammatory response [15].

In our study, COVID-19 mortality was associated with four biomarkers: serum albumin, direct bilirubin, total leucocyte count, and respiratory rate. Serum albumin has been proven to be an acute phase reactant. Under conditions of increased oxidative stress, it can undergo irreversible oxidation leading to increased cellular injury [19]. Patients who were severely affected, admitted to ICU, and those who required ventilatory support reported to have lower serum albumin levels than the mild to moderately affected population (3.37 vs 3.83, p = 0.013). Bilirubin levels are reflective of acute liver injury, and direct bilirubin was increased among the nonsurvivors of our study population (0.29 vs 1.19 p = 0.001). Chai et al. [20] showed that the expression of ACE-2 receptors on cholangiocytes was equivalent to that of the type II alveolar cells; however, the number is 20 times less in the hepatocytes. A study done in China by Liu et al. also showed that total bilirubin (HR: 9.45, 95% confidence interval (CI): 2.21-40.47, p = 0.002), direct bilirubin levels (HR: 4.38, 95% CI: 1.78-16.29, p = 0.03), and conjugated bilirubin/unconjugated bilirubin (CB/UCB) (HR: 2.49, 95% CI: 1.32-4.71, p = 0.01) were higher in the nonsurviving group [21] signifying toward an important mechanism of acute liver injury. COVID-19 being an infection triggers the inflammatory cascade and cytokines such as IL-6, IL-17, and TH-17, leading to SIRS, ARDS, and multiorgan failure. Hence the reason why increased total leucocyte count was seen as a marker influencing mortality (9.82 vs 13.37 p < 0.001). A study done in Wuhan, China, by Feng et al. also showed significant concordance between mortality and leukocytosis (HR = 1.14, 95% CI: 1.09-1.20, p < 0.001) [22]. As patients commonly complain about shortness of breath, patients more severely affected with COVID-19 had higher incidences of respiratory failure as suggested by the higher respiratory rate (22.75 vs 25.59, p = 0.002), which was reflective of higher mortality in our study. Many studies globally showed similar results as well [23].

There are several strengths to this study. First, this study reflects the association between liver derangement and COVID-19 severity, as seen by the deranged LFTs along with COVID-19 inflammatory markers. Second, in this study by removing patients with preexisting and/or newly diagnosed chronic liver disease, we were able to see how COVID-19 affects naïve liver and see the clinical course. Lastly, this study also substantiates the use of AST, GGT, and albumin as COVID-19 severity markers as well as albumin and direct bilirubin as mortality markers along with raised leucocyte count and tachypnoea.

The limitations of this study are as follows: a sample size of 135 cases is smaller as compared to other studies that have been conducted but is still relevant to show a distinct derangement in liver enzyme levels. Furthermore, the cases were all from a single hospital in a certain demographic of Pakistan, which may not show a proper representation of the disease and its effect on liver enzymes on a country-wide basis. Lastly, the laboratory results that were used in this study were results seen on admission, and so the effect of the progress on said laboratory results during a hospital stay has not been utilized.

## Conclusions

In conclusion, COVID-19 has multisystemic effects. Liver injury caused by COVID-19 stems majorly from inflammatory response due to cytokines. The degree of liver damage is reflective of the severity and increased mortality, hence showing evidence that liver function enzymes have the potential to be utilized as surrogates to dictate the prognosis of COVID-19 severity.
